# What parents want in an autism diagnostic report: An interview-based study of parents accessing a neurodevelopmental assessment service

**DOI:** 10.1177/13591045221138703

**Published:** 2022-11-14

**Authors:** Alexander C Wilson, Stef Gunn

**Affiliations:** 1 School of Psychology, 6396 Newcastle University, Newcastle upon Tyne, UK; 2 Neurodevelopmental Assessment Service for Durham and Darlington, Stanley Primary Care Centre, 3059 Tees Esk and Wear Valleys NHS Foundation Trust, Durham, UK

**Keywords:** autism, assessment, diagnosis, report, neurodevelopmental conditions

## Abstract

Diagnostic reports are a key outcome of autism assessment services. However, there is limited evidence regarding what key stakeholders, including families, want to see in reports. In this project, 30 parents whose young person had recently received a diagnosis of autism from a Neurodevelopmental Assessment Service in the North East of England took part in a telephone-based interview to explore what they want from a report. Interviews were analysed using thematic analysis. Ten key recommendations for reports were identified. Parents indicated that they want a detailed, balanced, sensitively written report. They highlighted that reports needed to be accessible and clearly structured. In this respect, it might be helpful to include a parent-driven summary of key points at the top, clear signposting of the structure of the report, and a description of what happened in the assessment process. Parents also valued practical, personalised recommendations based on the young person’s strengths and difficulties. Future research might explore perspectives on reports in families accessing other services, in other client groups (e.g., families of pre-schoolers diagnosed with autism), and with different stakeholders, including schools, referrers and autistic people.

## Introduction

Clinicians hold a significant responsibility when sharing with young people and their families a report about the young person’s autism diagnosis. A diagnosis is likely to influence a person’s identity and how others understand them. It may also tell us about the person’s needs now and in the future, so clinicians may feel a need to “get it right” when putting together a report. From the family perspective, the report is an important document, as satisfaction with the report strongly predicts how satisfied families are with the overall assessment process (Eggleston et al., 2019). However, the evidence base is limited regarding what young people and their families want in a diagnostic report. A service evaluation took place within an NHS-based Neurodevelopmental Assessment Service to learn about parents’ views regarding diagnostic reports.

This project was based in the Neurodevelopmental Assessment Service within Durham and Darlington Child and Adolescent Mental Health Services (CAMHS) in the United Kingdom. Referrals come via the CAMHS single point of access service on behalf of any young person in the local area where there is a query over neurodevelopmental differences. The service works to a week-long assessment model where most young people referred for an autism assessment have the majority of their assessment appointments over a 5 day period. This assessment week includes sessions with the young person and their family, a multidisciplinary diagnostic meeting to discuss the information gathered against ICD-10 criteria for “childhood autism” (World Health Organisation, 1993), and a feedback session to share the outcome of the assessment with the family. Diagnostic reports are typically put together “live” during the diagnostic meeting. The reports collate information from a range of sources including direct observation of the young person, the developmental history shared by the family, information from school, and information from other professionals. Reports evaluated in this project were written to the following structure: they stated the outcome of the assessment and gave the young person’s early history, then described the young person’s current presentation in relation to the diagnostic criteria for autism, and ended with recommendations. The main body of the report included tables for each of the ICD-10 criteria. In each section, (1) the criterion was given, then (2) detailed evidence relating to the criterion was quoted from reports from each assessment appointment/setting, and then (3) a consensus statement concluded whether the young person met that criterion. This structure was repeated for all criteria.

The National Institute for Health and Care Excellence (NICE, 2011) provides some guidance about reports in Clinical Guideline 128 *Autism spectrum disorder in under 19s: recognition, referral and diagnosis* [CG128 1.8]. The guideline indicates that the report should explain the findings of the assessment and reasons for conclusions drawn; be “sensitive”; and refer to the young person’s “profile”. The guideline also indicates that findings may be shared, if appropriate, with the child, and subject to the family’s consent, with relevant education and social care professionals too, so it can contribute to the young person’s individual education plan and needs-based management plan. This assumes the report is accessible and useful to these groups. We set out to assess how well reports met these principles, according to parents.

In line with person-centred practice, we also wanted to explore more generally how parents found reports and whether they had suggestions for things that could be done differently. There is a limited number of previous studies relevant to these issues, but existing literature gives some insight into what families might look for. In a small qualitative study, Abbott et al. (2013) found that families appreciated feedback to be structured, holistic and hopeful, with a balanced account of a child’s strengths and difficulties. In addition, Hennel et al. (2016) suggested that a tailored included a tailored ‘autism action plan’ (e.g., with recommendations) could improve satisfaction with reports. It should be noted that although parent and child satisfaction is vitally important, we do need to be mindful about other factors too, when evaluating reports. These might include service pressures (limited resources, waiting lists, etc.), as well as the needs of other audiences (e.g., schools and clinicians in other services who might be working with the young person). Our project did not directly focus on these issues, but they should be kept in mind when evaluating the study described below and we offer some reflections at the end.

Our evaluation of diagnostic reports came at a timely point for our service. The team was transitioning from a service structure with separate pathways for autism and ADHD to a needs-led neurodevelopmental pathway. The team was keen to develop a new type of report to fit the changes to the service, but it was important not to lose elements of the reports that families appreciated. This service evaluation project therefore fed into an action plan to optimise our report-writing. Our broader aim was to add to the evidence base around report-writing in a person-centred way, providing some guidance from parents about what they want to see in reports.

## Method

Trust approval for this project was granted by the Clinical Audit and Effectiveness Steering Group, Tees, Esk and Wear Valleys NHS Foundation Trust in August 2021 [ref. 6591CAMHS21].

### Participants

Thirty parents were informed about the project, and all consented to take part, either in a phone interview (*N* = 27) or to complete a written questionnaire (*N* = 3). These were parents of young people aged between 6 and 17 years (Mean = 10 years; 10 months; SD = 3 years; 6 months). Nineteen young people were male and 11 were female. Four young people had a co-occurring diagnosis alongside autism, and all others had a single diagnosis of autism. All young people communicated in sentence level speech and were assessed or estimated to have cognitive ability in the typical range. All diagnoses of autism were made within our team between May and September 2021. Families lived across County Durham in the UK. As an indication of regional social economic status, County Durham received an Index of Deprivation in the fourth decile (Hewitt & Surtees, 2020).

### Procedure

Parents whose young person had received a diagnosis in the service between May and September 2021 were contacted in consecutive order until 30 parents consented to take part. 10 parents were not contactable, but all parents who answered the phone consented to share some views/feedback about the diagnostic report they had received, either answering questions over the phone or completing a written questionnaire. Verbal consent was gained for phone interviews, whereas written consent was sought if parents preferred to complete the questionnaire. During phone interviews, parents were asked how their young person and wider family were doing since the autism diagnosis, and any questions/queries were explored with families. Parents then completed a short interview where they shared their perspectives on the diagnostic report they had received. This took about 5–10 minutes. All phone interviews were carried out by the first author (ACW) who took detailed notes to record parents' comments in their own words.

### Materials

Interviews and the written questionnaire followed the same structure. First, families were asked to rate on a 7-point Likert scale how satisfied they were (1) with the assessment process and (2) specifically with the report. There were then 16 questions asking about different aspects of the report (e.g., coverage of strengths and difficulties, length, recommendations, accessibility to different people). Each question was answered by yes, no or partly/maybe for ease of administration, but families were invited to elaborate wherever they wanted. At the end, families were asked for any more general comments and whether there was anything about the report they would change, remove or add. See Appendix for the full questionnaire.

The questions were put together by the authors in consultation with the wider team to identify what clinicians wanted to know from families. We also ensured questions covered the key qualities and features suggested by NICE for autism diagnostic reports so that we could assess whether our reports complied with the guideline.

### Analysis

We computed descriptive statistics across questions that gave quantitative data, and carried out thematic analysis of the qualitative comments. Thematic analysis followed the practical guidelines set out by Braun and Clarke (2006). In an initial stage of thematic analysis, we read through participants’ comments to become familiar with the views expressed. Then we carried out an initial coding of the data; wherever a participant expressed something about the reports that represented a unit of information, this was coded. Initial codes kept to participants’ words as far as possible, with over 150 individual codes in total. In a next stage of coding, initial codes with a similar meaning were renamed using the same code – what we called the ‘first-order code’. First-order codes sharing a similar theme were then grouped under a second-order code to summarise that information.

## Results

24 (80%) of families said they were ‘very satisfied’ with the assessment process, and 6 (20%) reported being ‘satisfied’. Specifically regarding the report, 19 (63%) families described themselves as ‘very satisfied’, 10 (33%) reported being ‘satisfied’, and 1 (4%) said they were ‘neither satisfied nor dissatisfied’. Quantitative data for all other questions are summarised in Table 1.

**Table 1.** Table showing quantitative responses to questions about the diagnostic reports. An abbreviated form of each question has been given; the full version can be seen in the appendix. Frequencies (percentages) are shown in the columns.

Question	Yes	Maybe/Partly	No
Clear what the outcome was?	29 (97)	0 (0)	1 (3)
Understand how we did the assessment?	26 (87)	4 (13)	0 (0)
Written with families in mind?	25 (83)	5 (17)	0 (0)
Respectful and sensitive?	29 (97)	1 (3)	0 (0)
Okay seeing your/your young person’s words in report?	29 (97)	1 (3)	0 (0)
Clear wording?	27 (90)	3 (10)	0 (0)
Understand terminology?	26 (87)	3 (10)	1 (3)
Structure clear and easy to follow?	25 (83)	4 (13)	1 (3)
Good length?	30 (100)	0 (0)	0 (0)
Good amount of detail?	30 (100)	0 (0)	0 (0)
Full and accurate description of child’s difficulties?	26 (87)	4 (13)	0 (0)
Pay enough attention to strengths?	28 (93)	2 (7)	0 (0)
Enough recommendations?	22 (73)	8 (27)	0 (0)
Useful recommendations?	23 (77)	7 (23)	0 (0)
Report useful to professionals?	25 (83)	5 (17)	0 (0)
Report suitable for sharing with young person?	22 (73)	6 (20)	2 (7)

Table 2 shows a summary of our thematic analysis, and below we discuss each of the key points raised by this analysis.

**Table 2.** Qualitative analysis of feedback given by families. First-order codes are given in normal type and second-order codes in larger, bold type. First-order codes are categorised as strengths (green) and constructive feedback/practical ideas for improvement (orange).

**1/Families want a lengthy, detailed report**
The report is comprehensive
It documents what was found
It needs to be long
The length allows you to trust the outcome
The longer the better
The length might be difficult for other people with less “insider” knowledge
**2/Families want a sensitive report that reflects the young person on their own terms**
It is good to include the parent’s and young person’s own words
This is important in feeling listened to and understood
The report shows thoughtfulness about how the parent would feel reading it
It provides a personalised description
It gives an accurate depiction of the young person – as if you “got” them
**3/Families want a report that celebrates strengths**
The report is holistic
It celebrates strengths
**4/Families want the report to be clear and accessible in its structure and language**
The report is accessible
There is a clear structure with sections organised by criteria and setting
The report relies on some existing knowledge from the parent about criteria/terminology
It is not fully accessible to families (as it caters for multiple audiences)
It is not always clear or easy to follow the different sections
It may be less appropriate to neurodiverse people (including neurodiverse parents)
It would be good to have a parent-driven summary that is simpler and softer covering key features and recommendations at the beginning
It may benefit from professional support to digest
**5/Families want a report that is clear and direct but tactful**
The report has a clear meaning
It is good to have a direct and unambiguous approach
The report can be emotive due to the frank discussion of difficulties
There were some blunt words from the parent/school included in the report
It can be draining to read where the tone is impersonal
**6/Families want a report/version of the report for their young person**
The young person deserves to see the report as they are the subject of it
The report is mostly accessible to the young person as it is (depending on the young person’s age and the nature of their difficulties, as well as possible contextual/timing issues)
It allows more understanding about oneself
It is not always appropriate for the young person due to some negative content
It may be useful to have a simpler, softer version for the young person
**7/Families want to understand the diagnostic process behind the report**
The report documents the assessment process
It is important to be transparent about what happened in the appointments
It is not always clear from the report how the assessment was done
It is not always clear how the criteria work
An introduction to the criteria would improve clarity
**8/Families want a report that shares information across settings**
The report includes information from multiple sources
It brings people together in the best interests of the young person
The report supports others to understand the young person (especially school)
It allows communication about the young person’s needs to others
It gives families greater insight into what is happening in school
There is not always enough coverage of all settings (especially school)
**9/Families want a report that gives something concrete to reflect on**
The report gives a new understanding for the parent
It stimulates further thought and discussion
It allows the family some closure
**10/Families want a report that gives meaningful recommendations**
The report sets expectations for support
The report is sufficient to support the young person’s development (without adding lots of extra recommendations)
It provides useful signposting to organisations
There are useful recommendations for things you can do
The report explains the meaning of the diagnosis for support
There are not enough (targeted/personalised) recommendations
There is an overwhelming number of signposting links given

### Families Want a Lengthy, Detailed Report

Parents/Carers consistently said they appreciated a detailed account of their young person’s autistic traits, and that the length of the report helped them have confidence in the outcome. As one family commented, “It was good how it went in depth on every part, I found it really helpful so I could trust it and know you’d done your job [...] It described [X’s] difficulties completely, everything was bang on about [X] from the professionals throughout the report”. Likewise, another family noted, “It’s a big thing, a diagnosis, so you need that length to see how it was concluded”. Occasionally families commented that a very lengthy report could potentially be confusing, but the consensus was that a longer report was preferred. Families generally agreed that “It would never be too long […] the more information the better”.

### Families Want a Sensitive Report that Reflects their Young Person on their Own Terms

Several parents/carers spoke with appreciation that the report was their young person “to a T”. In part, they linked this to the direct inclusion of the young person’s and family’s own words in the report. One family said, “I loved the bits where you added words [X] used. It felt honest and like he had been listened to. The child’s words haven’t been turned to mean something else, it proves they’ve listened to the child and not interpreted it into something else”. Another family felt similarly: “It was really personal and specific to [X], it was really good where you put in things he actually said in the appointment, like where he corrected someone in the assessment, I can just imagine him saying that, by quoting him in the report it gave me confidence you had listened to him and got him”. Parents commented on their own experiences of feeling listened to and considered by clinicians; e.g. “In the report you can see that my point of view is really listened to and taken into the report […] And the way it’s written is careful, they’ve taken a lot of things into consideration and thought about how I’d feel reading it as a parent and it’s very respectful how they’ve done it.”

### Families Want a Report that Celebrates their Young Person’s Strengths

One parent commented, “Autism brings some really special stuff, it’s really nice to talk about what he shines at and see that reflected in the report […] There’s not much opportunity for you to hear about all the things he shines at. It’s nice to focus on interests, and in a really positive way that doesn’t see them as a problem, like seeing his trains as something great about him rather than a quirky thing”. Discussing strengths allows the report a more hopeful, celebratory tone. As one family said, “Definitely there were strengths mentioned in the report. It gave us lots as parents to arm ourselves with in putting a positive mind-set on it all”.

### Families Want the Report to be Clear and Accessible in its Structure and Language

This need was often met by the reports. For instance, one parent commented, “It’s written in really clear and accessible language, I could see the effort was there to do that [...] It’s all set out really clearly with all the criteria and it was clear whether she had met each, it was great to see the evidence from each party set out so clearly”. Another family shared a similar view, “They put it in their own words so you could understand everything, it read like something someone just wrote normally, not really long words, it was understandable by me and [X]”. However, not all parents agreed, as some parents felt you needed to have “insider knowledge” in order to understand the report in full. One parent noted, “I think it was probably easier for me to read as I work in learning disabilities. It’s just quite long and there’s lots of different bits to it. I think you kind of need to know what you’re looking at to get it”. One parent (possibly without this “insider knowledge”) shared the following: “The criteria were difficult to follow. I didn’t realise that it was split into separate sections for each criteria. It’s not obvious when you look at it what that means. And I thought it would all be the parent stuff then school or whatever rather than split into each criteria [...] It could have been a bit clearer, maybe having an introduction at the top so you understand how it’s set out.” As such, the structure of the report sometimes seemed more appropriate to professionals than families. Another parent made the same suggestion that we include an additional parent-driven summary at the top of the report to help orientate the family to what they are seeing: “The only thing that is a challenge is that the report is driven by several audiences – parents, school and GP. Maybe there could be a small summary that is more parent driven that is softer that is more simplified and says some of the nice things about the child as well as challenges and these are the things you could do to help.”

### Families Want a Report that is Clear and Direct but Tactful

One parent said, “There’s no beating around the bush. Sometimes teachers can be a bit vague, it’s direct and to the point, which I like, it’s clear what you meant.” Usually, this directness was taken well by families, but parents did comment that occasionally there was a blunt comment that was more difficult to read – often these comments had been directly lifted from reports sent in by school. One parent did seem to find the report difficult to read overall, commenting, “The tone of the report is impersonal. It is very draining to read, and expects the parent or carer to take on a lot of very condensed information alone, with no professional support.”

### Families Want a Report/Version of the Report for their Young Person

Parents often suggested that young people had a right to see a report as it was them in the report. One parent commented, “Our son might never understand what it means, but we’ve never lied to him and I’d want him to see the report. He needs to understand his story, it is part of his makeup, it might help him understand himself and his journey through his younger learning years.” Other parents also believed that a report might help the young person develop their self-awareness. For instance, one parent shared the following reason for showing their young person the report: “For her to see for herself. I think for her she doesn’t always recognise things for herself and she might like to see what others perceive about her”. Parents generally felt that the reports produced in the service were appropriate for their young people to read. For instance, one parent of a teenage son shared, “He was happy with reading the report, he took it in his stride. It might depend on their age and maybe their difficulties”. Other parents also agreed with this disclaimer: that the suitability of the report might depend on age and difficulties and other factors (although most shared a willingness and intention to share the report at some point). A small number of parents did say they probably would not share the report, and this was mostly due to negative content in the report. One parent commented, “It’s not something I would show him as he is really sensitive and takes things to heart. It’s a full report that focuses on what he can’t manage, you’ve got comments from teachers that wouldn’t be good for him to sit and read”. Likewise, another parent said, “It might be useful to write a version of the report that was more appropriate to share with the subject of the report. For example, reading their own name repeated endlessly and their unconventional behaviours described frankly might be upsetting.” Other parents agreed it might be helpful to have a separate report for the young person, either to simplify it or remove some of the less comfortable content.

### Families Want to Understand the Diagnostic Process Behind the Report

This was an area that some parents highlighted as somewhat lacking in the report: “I don’t think you would understand from the report itself how the assessment was done. But it was explained to me in the appointment with the clinician and I understood it from my own experience working in special needs rather than the report itself.” Parents felt that it was important that the report explained what happened during the assessment (and not just the outcome). They appreciated transparency and openness and having a full record of a process that marked an important milestone in understanding and supporting their young person. One parent said, “It explained what happened in the room as I couldn’t go in there, and explained everything so it was like I had been in there and could see the outcome of that”.

### Families Want a Report that Shares Information Across Settings

The reports brought together multiple sources of information in the best interests of the young person. One parent noted, “The report was really helpful, great to see the evidence from all the different settings and from us, great to see how it all triangulated and met each of the criteria.” Seeing information from different settings often brought new understanding; several parents commented in particular about gaining additional insights about school from the report. Typically, parents learnt one of two things: either that school was seeing difficulties that parents were previously unaware of, or that school was possibly missing difficulties. One parent shared, “We know he has some problems that they have not realised so we need to discuss that. I don’t think the teacher really understood him or noticed much. It’s a good insight that they haven’t always understood the difficulties.” Thinking about the future, parents also felt the report had an information-sharing role: “It helps the school understand, and it saves me having to explain everything all over again”.

### Families Want a Report that Gives Something Concrete to Reflect on

Many parents spoke about using the report to pause, process and understand their young person. One parent shared, “I can never read enough about my boy, anything that helps us support the development of our boy […] I needed to understand [X] and that has completely ticked the box.” Another parent particularly commented on how the report helped develop new perspectives for all family members: “You could sit down for 20 or 30 minutes to digest it as it’s a good length and take in all the information, it was really good for me to sit with it [...] It was good especially to see where we differed in opinion, it brought us together and see that all written there, what all the different people had seen. […] I think it has changed our perspective as a family for the better […] I think it was really respectful and highlighting the things she has done, the amazing job she has done despite the barriers she experiences, I think the assessment supercharged feelings of being proud because I’ve seen just how much she has had to get through […] And the assessment had that effect for [X] too. She’s moved away from what’s wrong with me to what can I do make an extra effort with this, what will help me have a better wellbeing, what things should I be wary of. She’s really thought about it and researched it.” Parents often alluded to a theme of closure; as one parent said, the report was “the extra bit we needed to move on.”

### Families Want a Report that Gives Meaningful Recommendations

Some parents felt that the report would prepare the ground for support. As one parent said, “Say he was moving to the comprehensive school, say the report was the first thing they saw, it would be clear in saying this child will need this. It will set up an expectation for what they will need.” However, sometimes parents said that, although the report did a good job of assessing where the young person was now, it did not always help proactively with the future. One parent shared, “It seemed like it was really just a description of [X]. There wasn’t that much we could try to support [X]. I’d have liked more of that”. Another parent agreed with this view: “When I got the diagnosis, I did feel a bit of what next. It feels here’s the diagnosis, here’s the names of the websites, go and have a look. Maybe you could have a follow-up phone call to talk about particular sources of help that might be most helpful to the child”. This parent seemed to want more personalised guidance around support. Likewise, another parent commented on needing more direct recommendations for school, as their young person experienced the most challenges there: “I think there could be a more comprehensive list of hints and tips to school. I’ve learnt a lot but it might not always be obvious to other people. There’s a lot we talked about on the phone that could be useful in a one pager.” With respect to recommendations, the reports focus on listing details about support organisations. This was often appreciated. One parent said, “I’ve got a lot of support ideas at the end [...] I’ve already contacted one of them and they’ve helped with my issues with the school.” However, other families felt the list of organisations was too generic or there were too many different organisations listed. One parent said, “I was shocked how you get a load of links to organisations. That can be overwhelming, I had researched a lot so it was okay for me but I wonder about other people, where would you even start?”

The consensus was that reports could go a little further in suggesting personalised recommendations. However, one parent did challenge this idea: “You want something to tell you here’s what you can do next but I’m not sure that is realistic. I know other parents say “they left me in the dark”, but you’ve parented your child, you know what works for them and you need to make those links in the light of a new diagnosis. I don’t think anyone else can tell you what to do […] But I do think it might be useful for you to help parents understand what the potential outcome could be of the assessment. Maybe something in the parent interview to say don’t expect a toolkit to deal with the autism diagnosis if that happens. You just get on with your life, there’s no magic wand. Maybe as clinicians you can give some structure around those expectations. I also think there needs to more clarity on what CAMHS is there for.” This comment highlights that we need to be clear what the purpose of the report is; for this parent, the purpose is understanding rather than providing a “tool-kit”. As clinicians, it might be important to have these discussions with individual families to understand what they might hope for from their particular report.

## Discussion

In this project, feedback was collected about diagnostic reports from parents of young people who had recently received an autism diagnosis. We aimed to understand what parents want from the diagnostic report, using NICE guidance as a starting point for understanding what a good report might include from the perspective of parents.

As described above, NICE Clinical Guideline 128 states that the report should explain the outcome of the assessment sensitively with reference to the young person’s profile. It is clear from Table 1 that generally the service is meeting these expectations from the perspective of families. The vast majority of parents indicated that reports clearly explained the outcome in a sensitive way with full and accurate description of difficulties and sufficient attention to strengths. The NICE guideline also indicates that the report should be shared where appropriate with professionals involved with the young person and with the young person themselves. Generally, parents felt the report would be useful to professionals, but were less sure about how appropriate the report was for their young person. Some parents indicated they might “maybe” share the report with their young person depending on age and the young person’s presentation, and occasionally parents felt some of the content was too negative to share. A couple of parents suggested that their young people might appreciate a slightly simpler, softer version of the report. Overall, parents felt strongly that young people deserved to see a report if they wanted – it was them in the report, so they had a right to.

There were a couple of issues where we as clinicians were uncertain what to expect from parental feedback. First, we were unsure what parents would make of the length of reports and wondered if they might prefer something shorter and more of a summary. However, this was not the case. Parents really appreciated a long report; no-one suggested that a shorter report would be better and the consensus was “the longer the better”. Parents highlighted that this level of detail helped them to trust the assessment process and the outcome, and was appropriate for an important document. In addition, we were unsure whether families would feel comfortable seeing their own and their young person’s words in the report. However, from parents there was no uncertainty. Each parents indicated they liked seeing their own words. This helped parents feel listened to, and showed that clinicians had captured the young person on their own terms.

Parents gave two key ideas for improving reports. Some parents highlighted that the report could sometimes be difficult to follow, and they felt that a parent-driven summary at the top would help. They suggested that this should include a brief summary of the child’s autistic traits, their strengths and brief suggestions of what you could do to support them. Parents also suggested giving a summary of the structure of the report and assessment itself (e.g., summarising the criteria used and what happened in the different appointments). This would also help orientate families to the report. The second suggestion made by parents to improve the reports was to be more personalised when making recommendations around support. It did not seem that parents wanted lengthy recommendations, but practical suggestions that might make little changes in areas posing the most challenge for their young person.

Overall, the key learning points from this project are consistent with the small amount of existing research. Abbott et al. (2013) found that families appreciated feedback to be structured, holistic and hopeful, with a balanced account of a child’s strengths and difficulties; this was very much what we found. In addition, Hennel et al. (2016) suggested that a tailored ‘autism action plan’ (with recommendations) could improve satisfaction with reports, and this is very similar to the point made above. Indeed, this suggestion regarding recommendations was the most common area for improvement flagged by families. It is worth thinking about the key learning points of this project in a broader context, given that we only directly focused on parent satisfaction here. We should also consider systemic issues and the perspectives of other target audiences, as these may sometimes conflict with parental preferences. For instance, services are typically working with limited resources and considerable waiting lists, and it may not be practical to produce extremely long reports if this affects how many families can be seen. Similarly, education professionals may prefer a briefer report more directly focussed on needs and difficulties, as this may make a clearer case for support in school. Ultimately, such a report may be in the best interests of a young person and could be written sensitively, but it may not be ideal in the eyes of families and young people themselves, who may prefer more celebration of strengths. Therefore, the practical implications of this study need to be considered with relation to the multiple functions and audiences of a report in order to serve young people in the best way.

In this project, we were able to hear views from a good number of parents that represented a random sample of those accessing the service. Every family approached consented to take part, indicating minimal risk of selection bias. Speaking to families over the phone allowed us to collect a richer dataset than if we had simply sent out a questionnaire to everyone – e.g., we were able to ask for elaboration and explore comments further. We are therefore confident in the quality of the data. However, there are some limitations. All families were seen in one service in the North East of England, and all young people were school-aged children with cognitive ability in the typical range. Therefore, it is unclear how well these results might generalise to other services/regions and families of younger children and those with a co-occurring learning disability. In addition, the project focussed purely on what parents made of the reports, as we have mentioned above. Therefore, we do not know the views of other relevant audiences, including autistic people themselves, referrers and educational services. Future research might seek to replicate this study in other services and groups. Lastly, this study was set up as a service evaluation rather than large-scale research, so the focus was limited to evaluating our own existing practice. This means that the project has relatively limited information to provide regarding aspects of practice not followed by our service. For instance, unlike our service, some assessment services may include quantitative results of standardised diagnostic instruments in reports, such as scores from the Autism Diagnostic Observation Schedule (ADOS-2; Lord et al., 2012). Services may be curious whether it is helpful to include these scores, but this study is unable to answer this question.

The current study investigated parental perspectives on autism diagnostic reports. We found that parents wanted a detailed, balanced, sensitively written report. They emphasised that reports needed to be accessible and clearly structured. In this respect, it might be helpful to include a parent-driven summary of strengths, difficulties and recommendations at the top of the report. Similarly, reports should clearly signpost their structure (i.e., what sections are there in the report, and what do they include) and give some description of what happened during the assessment process (i.e., what information contributed to the assessment and what individual appointments consisted of). Parents also wanted practical, personalised recommendations based on the young person’s strengths and difficulties. These parental wishes should be held in mind alongside other issues influencing reports, such as systemic factors (e.g., service pressures) and the needs of other audiences (e.g., education professionals). Overall, our feedback from parents both supports the guidance set out by NICE and builds on it, helping us understand what would make autism diagnostic reports increasingly acceptable to families.

Trust approval for this project was granted by the Clinical Audit and Effectiveness Steering Group, Tees, Esk and Wear Valleys NHS Foundation Trust in August 2021 [ref. 6591CAMHS21].
